# Therapeutic role of mesenchymal stem cells in neurogenesis for management of neurological disorders: a scientometric study to an in-depth review

**DOI:** 10.3389/fneur.2025.1588535

**Published:** 2025-06-16

**Authors:** Qian Wang, Weifeng Jiang, Yan Feng, Lin Li, Lisheng Chu, Yan Fang

**Affiliations:** ^1^Department of Physiology, Zhejiang Chinese Medical University, Hangzhou, China; ^2^Department of Gerontology, The Quzhou Affiliated Hospital of Wenzhou Medical University, Quzhou People's Hospital, Quzhou, China

**Keywords:** neurogenesis, stroke, Alzheimer’s disease, mesenchymal stem cells, scientometric

## Abstract

**Background:**

The biological process of neurogenesis involves the production of new and completely functional neurons in two specific regions of the brain: the ventricular-subventricular zone (V-SVZ) and the subgranular zone (SGZ) of the hippocampal dentate gyrus by neural stem cells (NSCs). Interfering with this mechanism harms the brain and may result in neurological disorders. Cell-based therapies are becoming recognized as optimal possibilities for facilitating neurogenesis. To comprehend the many processes and mechanisms of neurogenesis and the role of mesenchymal stem cells (MSCs) as active contributors to pathologic events influencing neurogenesis. We utilized the Web of Science (core collection) as the data source.

**Methods:**

The search was performed in the Web of Science core collection database until April 30, 2024, with the terms “Mesenchymal stem cells” as the title and “neurogenesis” as the topic.

**Results:**

A total of 407 papers about mesenchymal stem cells in neurogenesis published from 2004 to 2024 were retrieved. Further, we performed a bibliometric analysis of these publications, such as generating cooperation maps, co-citation analysis of journals and references, and cluster analysis of keywords. Next, we discussed the mechanism by which MSCs promote neurogenesis during the onset of Alzheimer’s disease (AD) and stroke diseases.

**Conclusion:**

Overall, three aspects primarily reflect the treatment of stroke with MSCs: neural circuit reconstruction, mitochondrial transfer, and extracellular vesicle transfer. The treatment of AD with MSCs is mainly reflected in the five aspects of inhibiting neuroinflammation, microglia changes, amyloid-*β* removal, functional recovery of autophagy, and blood–brain barrier (BBB) function recovery. Finally, we also made prospects for future research of MSCs.

## 1 Introduction

Neurogenesis is the biological process through which neural stem cells (NSCs) produce new and fully functional neurons in two specific areas of the brain: the ventricular-subventricular zone (V-SVZ) and the subgranular zone (SGZ) of the hippocampal dentate gyrus (1). This mechanism mediates neuronal plasticity, the maintenance of the central nervous system (CNS), and cognitive function (2). Disrupting this process damages the brain and can lead to neurological diseases (3) like Huntington’s disease (HD) and Parkinson’s disease (PD), as well as psychiatric diseases (4) like depression and schizophrenia. Cell-based remedies emerge as ideal candidates for promoting neurogenesis (5). MSCs are the most used stem cells in biological medical research, and they can also migrate to the sites of injury and inflammation (6). Notwithstanding the intriguing and significant study about the regulation of neurogenesis by MSCs, there are yet no systematic reviews on this subject. To comprehend the many processes and mechanisms of neurogenesis and the role of MSCs as active contributors to pathologic events influencing neurogenesis. We analyzed the literature related to neurogenesis in MSCs using scientometrics.

Scientometrics is a statistical and mathematical approach for forecasting future trends, calculating data correlations, and conducting retrospective analyses (7). Emphasizing quantification, it is an all-encompassing knowledge system that amalgamates statistics, mathematics, and bibliographies. Bibliometrics serves as a supplementary research method extensively utilized across all disciplines (7). These scientometric analyses encapsulate prevailing research focal points and suggest prospective avenues for a certain subject. Consequently, scientometric approaches were employed to investigate and identify the focal points of mesenchymal stem cells in neurogenesis in this work.

## 2 Scientometric study

### 2.1 Data and methods

#### 2.1.1 Search and screening of literature

**Figure 1:**
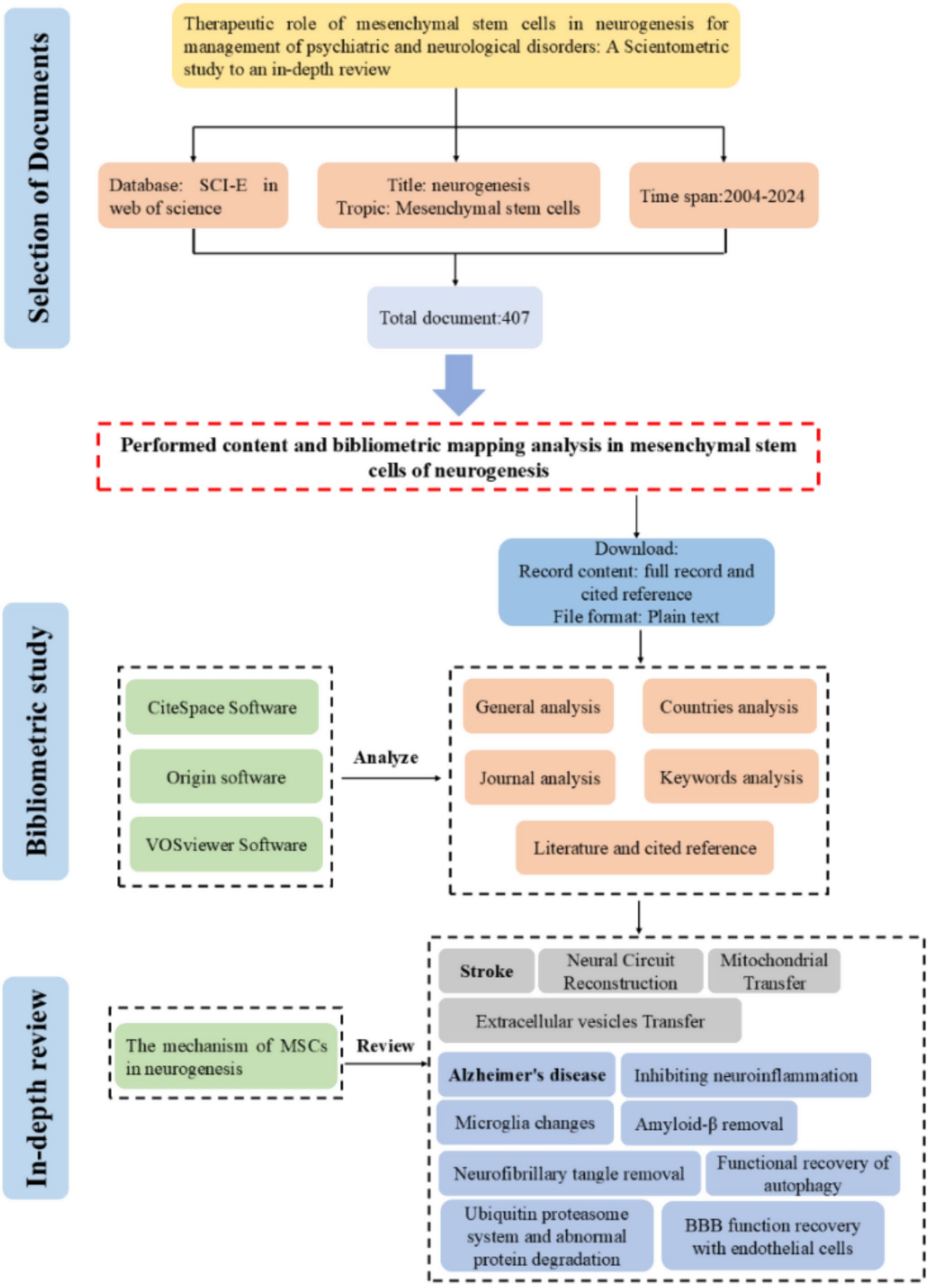
The workflow of study of MSCs on neurogenesis.

The search was conducted in the Web of Science core collection database until April 30, 2024, using the phrases “Mesenchymal stem cells” as the title and “neurogenesis” as the topic. We limited the sorts of literature to articles and reviews. No limits were imposed on language. The retrieved records were saved as plain text files and named download_txt. The flow chart of the paper is shown in Figure 1.

#### 2.1.2 Data download

The raw data were obtained from the Web of Science. The data had complete records and referenced citations. The file was formatted as plain text.

#### 2.1.3 Scientometric analysis and visualization

The raw data were analyzed using Bibliometrix (Version 3.13) in the R project (Version 4.1.0), as previously explained. Bibliometrix was used to extract the main information from the literature, including the publication number per year, nation or region, journal, keyword, and title. The data was visualized using R software and Origin software, respectively.

### 2.2 General analysis

**Figure 2:**
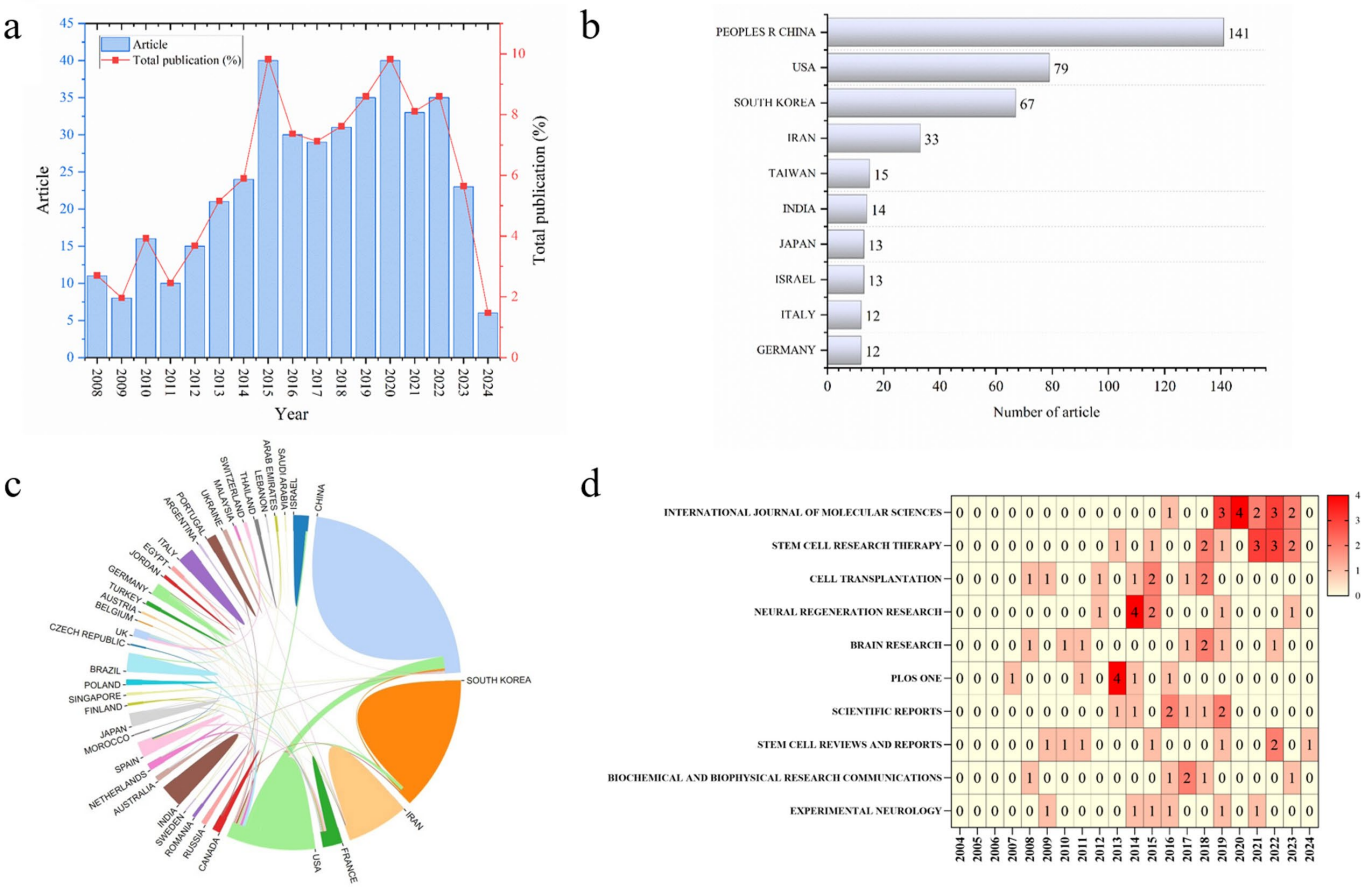
Scientometric study of MSCs on neurogenesis. **(a)** Number of publications by year. **(b)** Number of publications by country or region. **(c)** The cooperation relationships of country or region. **(d)** Number of publications by the journal.

A total of 407 publications on the therapeutic effects of MSCs on neurogenesis were obtained from the Web of Science core collection database prior to April 30, 2024. The average number of citations per document was 35.14. The first article on the therapeutic effect of MSCs on neurogenesis, which focuses on the bone marrow mesenchymal stem cells and Rett syndrome of apoptosis was published in 2008 (Figure 2a). Most articles were published in the year 2015 and 2020, while the most cited year was 2015. So far, research in the field of the therapeutic effect of MSCs on neurogenesis is increasingly ongoing.

### 2.3 Country/region analysis

A total of 46 countries/regions contributed to the publications included in this study. Among them, China published the most articles, followed by USA, South Korea, Iran, Taiwan, India, Japan, Israel, and Germany (Figure 2b). The partnership among countries demonstrated close cooperation between various countries (Figure 2c).

### 2.4 Journal analysis

All publications were published in 200 types of journals. The top 10 journals are shown in Figure 2d. The most relevant journal was “International journal of molecular sciences.” Stem cell research & therapy has the second highest volume of publications, followed by Cell transplantation, Neural regeneration research, Stem cell reviews and reports, Brain research, and Scientific reports.

### 2.5 Literature and cited reference

The top 10 most-cited papers about the therapeutic effect of MSCs on neurogenesis are shown in Table 1. The article with the most citations reported a long-term follow-up study of intravenous autologous MSCs transplantation in patients with ischemic stroke (8). The second most-cited article studied the effect of MSCs on angiogenesis and neurogenesis after cerebral ischemia in rats (9), as well as Parkinson’s disease (10) and neuroprotection from traumatic brain injury (11). The top 10 cited references on the therapeutic roles of MSCs in neurogenesis are shown in Table 2. These papers reported significant findings for researchers to discover the most influential studies in the field of therapeutic effect of MSCs on neurogenesis, further guiding in-depth research.

**Table 1:** The top 10 most-cited papers about the therapeutic effect of MSCs on neurogenesis.

Rank	Time cited	Journal			Impact factor (2022)	Ref
		Year	Name	Country or region		
1	587	2010	STEM CELLS	South of Korea	5.2	(8)
2	282	2012	NEUROBIOLOGY OF DISEASE	USA	6.1	(9)
3	282	2010	TRANSLATIONAL RESEARCH	India	7.8	(10)
4	262	2017	NEUROCHEMISTRY INTERNATIONAL	USA	4.2	(11)
5	230	2013	PLOS ONE	Taiwan	3.7	(92)
6	226	2010	BIOMATERIALS	Singapore	14	(93)
7	210	2013	CLINICAL SCIENCE	Taiwan	6	(94)
8	195	2010	BRAIN BEHAVIOR AND IMMUNITY	Netherlands	15.1	(95)
9	174	2008	BRAIN RESEARCH	South Korea	2.9	(96)
10	169	2011	BRAIN RESEARCH	Peoples R China	2.9	(21)

**Table 2:** The top 10 cited references about the therapeutic effect of MSCs on neurogenesis.

Rank	Time cited	Journal			Impact factor (2022)	Ref
		Year	Name	Country or region		
1	42	1999	SCIENCE	USA	56.9	(97)
2	35	2006	JOURNAL OF NEUROSCIENCE RESEARCH	USA	2.9	(98)
3	33	2006	CYTOTHERAY	Norway	4.5	(17)
4	33	2002	LANCET NEUROL	UK	30	(99)
5	31	2001	STROKE	USA	8.3	(100)
6	26	2000	EXPERIMENTAL NEUROLOGY	USA	5.3	(101)
7	24	2012	NEUROBIOLOGY Of DISEASE	ENGLAND	6.1	(102)
8	24	2003	JOURNAL OF NEUROSCIENCE RESEARC	USA	4.3	(103)
9	23	2002	NEUROLOGY	USA	9.9	(104)
10	23	2015	ANNALS Of NEUROLOGY	USA	11.2	(105)

### 2.6 Keyword analysis

**Figure 3:**
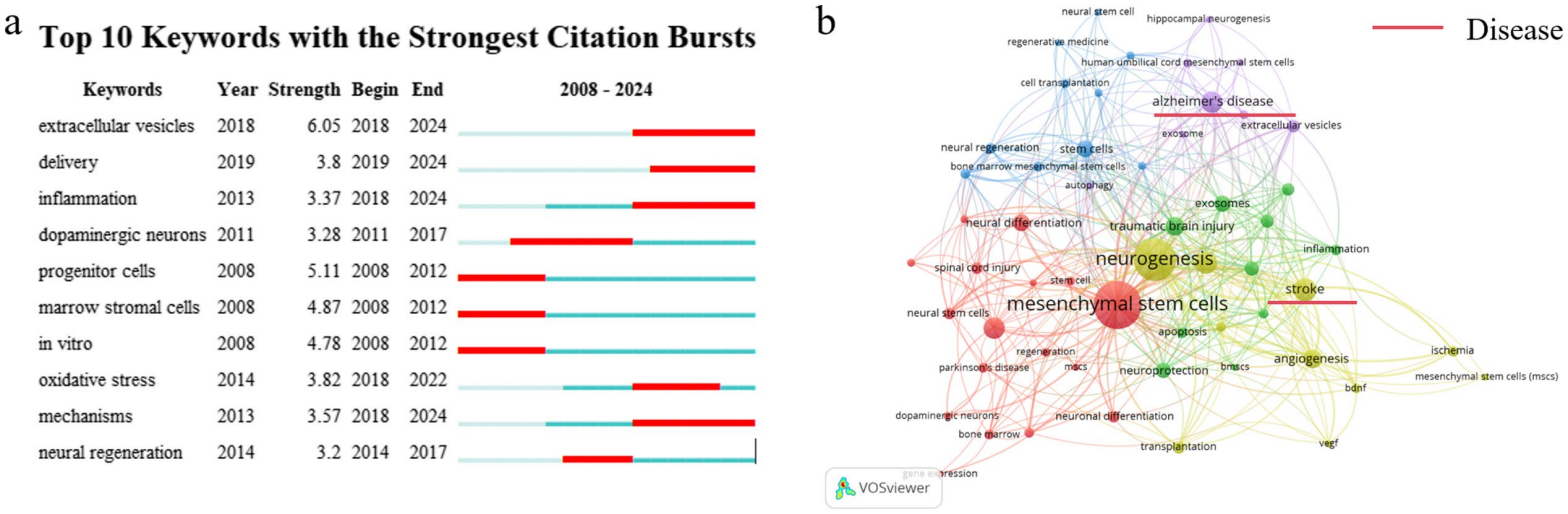
Keyword of analysis. **(a)** Top 10 keywords with strongest citation bursts. **(b)** Keyword co-occurrence diagram from 2008 to 2024.

According to the scientometric theory, keywords are indicative of current study trends and focal points within a specific research topic (12). In this review, keyword visualization was presented as keywords with citation bursts (Figure 3a) and a co-occurrence network (Figure 3b). Keywords exhibiting citation bursts imply that certain keywords have a higher number of citations within a specific timeframe. This phenomenon can be used to evaluate the level of interest in a particular study subject during that period and to identify developing ideas (13). Through the observation of keywords such as extracellular vesicles (strength, 6.05; time span, 2018–2024), delivery (strength, 3.8; time span, 2019–2024), inflammation (strength, 3.37; time span, 2013–2024), dopaminergic neurons (strength, 3.28; time span, 2011–2017) and progenitor cells (strength, 5.11; time span, 2008–2012) (Figure 3a), it is inferred that extracellular vesicles was a hotspot in the first ten years. In addition, the study of progenitor neurons by MSCs is also a hot topic. These MSCs’ mechanisms for neurogenesis focused on inflammation and oxidative stress. The co-occurrence network involved categorizing keywords into distinct clusters, each assigned a different color, based on their connection. Remarkably, “cognition” was a crossed keyword in these clusters, suggesting that MSCs may affect cognition. Stroke, traumatic brain injury, Parkinson’s disease and Alzheimer’s disease were among the CNS disorders whose keywords appeared in the co-occurrence network, as Figure 3b illustrates. We speculated that by regulating neurogenesis, MSCs would be beneficial to CNS disorders. Thus, we searched for articles on MSCs treating CNS diseases by regulating neurogenesis. Consequently, we analyzed the diseases involved in these 407 articles and finally found the most articles related to stroke and AD.

## 3 The mechanism of MSCs in neurogenesis

**Figure 4:**
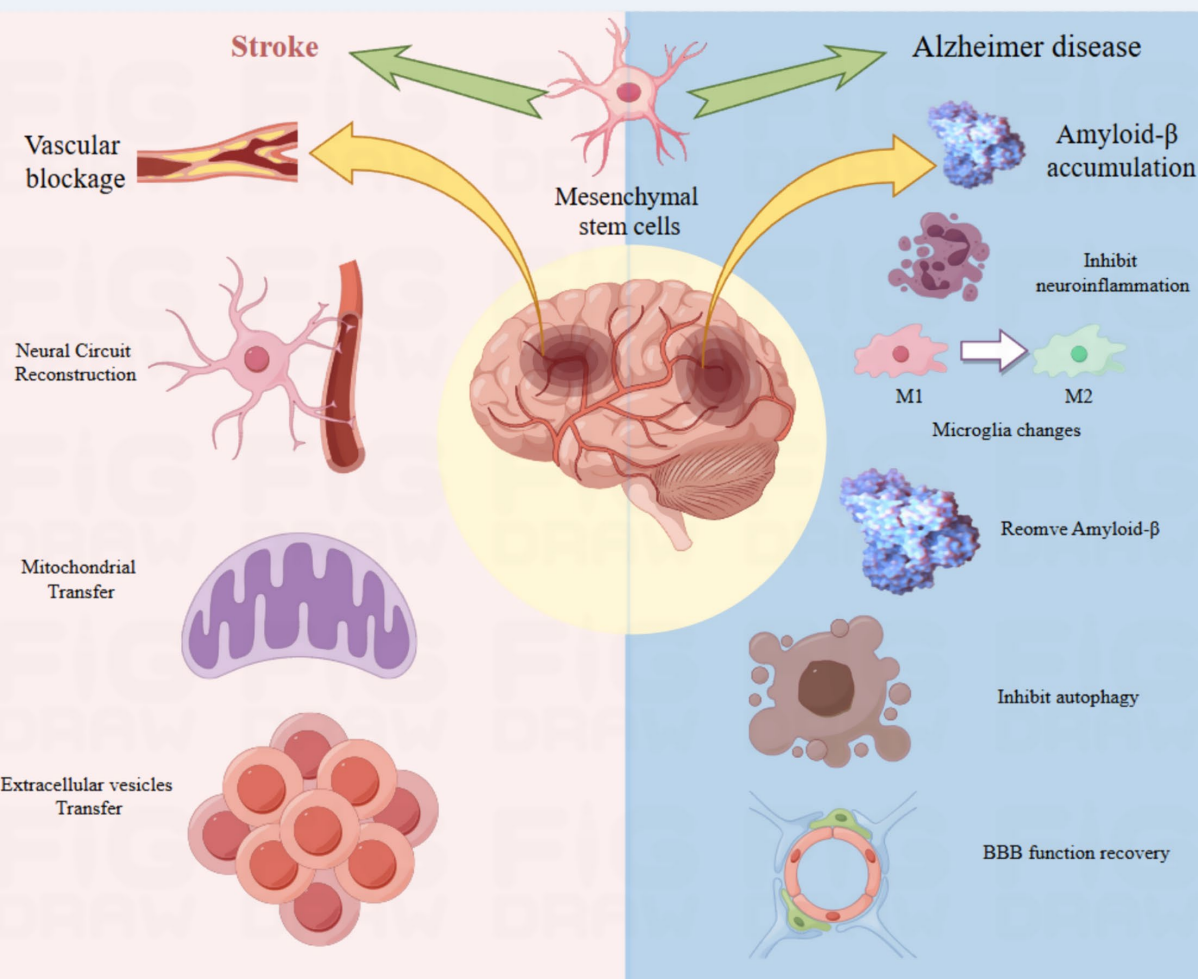
Scheme illustrates the mechanism of MSCs in neurogenesis.

MSCs are very attractive candidates for regenerative medicine owing to their excellent accessibility, *in vivo* proliferative capacity, and unique immunogenic properties. Moreover, these multipotent cells demonstrate remarkable environmental adaptability and secretory proficiency. Significant attention has been directed towards the ability of MSCs to move and assimilate into diverse tissues. Our bibliometric study delineated the processes through which MSCs contribute to the treatment of AD and stroke. The mechanism by which MSCs operate in the treatment of stroke and Alzheimer’s disease is illustrated in Figure 4.

### 3.1 MSCs and stroke

Stroke is a critical cerebrovascular disorder resulting from a disruption in cerebral blood flow, resulting in neurological and cognitive deficits (14). Stroke enhances the proliferation of NSCs and facilitates the migration of newly formed neuroblasts from the SVZ to the area surrounding the ischemic boundary. Moreover, stroke promotes the transformation of these neuroblasts into fully developed neurons (15). The findings suggest that fostering neurogenesis in the brain may serve as a potential target for rehabilitative therapy in individuals with ischemic brain injury (15). Recent studies have demonstrated that the administration of MSCs can diminish the extent of cerebral infarction following ischemia and facilitate the restoration of brain function (16). However, the number and replacement capacity of new neurons produced by the body itself after stroke are limited.

#### 3.1.1 Neural circuit reconstruction

Under suitable conditions, MSCs could differentiate into glial and neuronal cells (17). However, MSCs injected into the cortex surrounding the infarcted region can produce markers specific to neurons, the differentiated neurons are still young, spherical in shape, and have few fiber processes (18). More importantly, they lack the voltage-gated ion channels needed to generate action potentials (19). Therefore, the neural replacement mechanism may not be one of the mechanisms of MSCs in the treatment of ischemic stroke (IS). Endogenous neurogenesis takes place in the hippocampus’s SVZ and SGZ following cerebral ischemia. The recently discovered neural progenitor cells can go towards the damaged area caused by an infarction and undergo further specialization into neurons (20). Unfortunately, most of these neural progenitor cells are prone to rapid death due to the presence of inflammatory mediators and insufficient nourishment following ischemia. This hampers the injured area’s capacity to regenerate its neural network (20). It is reported that MSCs can increase the number of neural progenitor cells and promote endogenous neurogenesis after IS (21, 22). Song et al. observed that mice treated with MSCs had significantly increased neural network activity in the peri-infarct cortex, as determined by electrophysiological recording of evoked field potentials (22). Further investigation has shown that MSCs could improve the movement and survival of neuroblasts towards the area around an ischemia event, as well as increase the number of neurons in that same area (22). The upregulation of SDF-1 and polysialylation enzyme by MSCs facilitates the enhanced migration of neuroblasts towards the site of injury (23).

MSC treatment can weaken the physical and chemical barrier effect of glial scars on axonal regeneration after infarction. MSCs have the potential to increase the interhemispheric and intracortical axonal connections in the motor cortex around the infarction (24). Further research indicated that MSCs could support the rebuilding of neuronal connections by decreasing the thickness of glial scarring and the expression of Nogo-A (24). Furthermore, MSCs transplanted into the lesion may promote axonal growth by downregulating neuron expression and upregulating tPA expression in reactive astrocytes in glial scars (25–27). Transplanted MSCs may potentially enhance axonal development following brain ischemia by releasing nutrients. In mice treated with MSC, the cortex surrounding the infarction revealed an increase in GAP-43 expression, whereas axon growth inhibitory proteins Rock II and NG2 expression was decreased (28). It has been reported that transplanted MSCs can increase the number of oligodendrocyte progenitor cells in the peri-infarct area, corpus callosum, and SVZ (29, 30). The total amount of myelin basic protein increased significantly after MSC treatment, according to the researcher’s measurement of the protein in ipsilateral hemisphere tissue lysates of middle cerebral artery occlusion (MCAO) rats. This research indicates MSCs play a role in stimulating myelin production as well as neuronal circuit rebuilding (31).

#### 3.1.2 Mitochondrial transfer

MSCs could transfer healthy mitochondria to injured cells, potentially treating ischemic stroke. Tunnel nanotubes (TNTs) are tiny tubular structures at the nanoscale that link neighboring cells (32). As a novel method of communication between cells, they facilitate the transfer of cellular components between neighboring cells (33). Liu et al. found that TNTs between MSCs and endothelial cells developed when co-culturally MSCs with human umbilical vein endothelial cells exposed to oxygen glucose deprivation and reoxygenation (OGD/R). Furthermore, when exposed to OGD/R, the functioning mitochondria in MSCs migrate towards endothelial cells in a unidirectional manner, thereby safeguarding the endothelial cells from damage caused by hypoxia (34). The author demonstrated that the transplantation of MSCs following an ischemic stroke can offer protection to cerebral vascular endothelial cells via this intercellular connection. Their empirical research shows that when MSCs are transplanted into the area surrounding the peri-infarct region, they can transmit their functional mitochondria to the damaged microvascular endothelial cells. This procedure promotes the development of new blood vessels, reduces the size of the tissue affected by the infarction, and improves neurological function (35). Furthermore, the use of TNT inhibitors successfully prevented this behavior, suggesting that TNTs play a vital role in the transmission of this activity within mitochondria (35).

MSCs can transfer mitochondria to vascular endothelial cells, as well as to astrocytes and neurons that have been damaged by oxidative stress. This transfer of mitochondria helps to enhance the survival and proliferative growth of these cells (36, 37). The positive impact is contingent upon the direct interaction between cells, as the viability of neurons declined when MSCs and neurons were isolated by a permeable transmembrane (37). Moreover, it was observed that Miro1, a particular variant of Rho-GTPase located in mitochondria, exhibited an elevation in neurons that suffered oxidative damage. This rise in Miro1 promoted the transportation of mitochondria from MSCs to neurons (36). The researchers found that an increased quantity of neurons remained alive when they cultured Miro1 overexpressing MSCs with injured neurons. Conversely, Miro1 inhibition of MSCs resulted in the opposite outcome (37). Further *in vivo* experiments revealed that the transplantation of MSCs overexpressing Miro1 into rats with cerebral infarction resulted in a significant improvement in neuronal function compared to the transplantation of normal MSCs (37). In summary, after an ischemic stroke, the upregulation of Miro1 in neurons can lead to the transmission of functional mitochondria from transplanted MSCs to injured neurons. This process ultimately improves the metabolic function or viability of neurons.

#### 3.1.3 Extracellular vesicles transfer

Mesenchymal stem cell-derived extracellular vesicles (MSC-EVs) are spherical cytoplasmic components secreted by mesenchymal stem cells, which contain many soluble bioactive components such as lipids, proteins, mRNAs, and microRNAs (38). MSC-EVs serve a crucial role as important messengers between MSCs and damaged cells in the therapy of ischemic stroke. Xin et al. injected rats with MSCs-EVs via the tail vein 24 h after the induction of ischemic stroke (39). In comparison to the control group, the treatment group exhibited an increase in the density of axons and synaptophysin immunoreactive regions (39). Zhao et al. showed that the administration of exosomes produced from MSCs through intravenous injection, 2 h after an ischemic stroke, resulted in a significant decrease in neurological severity score and a notable improvement in motor function after 7 days (40). *In vitro*, exosomes derived from MSCs were co-cultured with OGD microglia. Researchers found that this might stop the activation of M1 microglia, increase the number of M2 microglia, lower the levels of cytokines that cause inflammation (TNF-*α*, IL-1*β*, and IL-12), and raise the levels of cytokines that prevent inflammation (TGF-β and IL-10) (40).

Furthermore, MSC-EVs may play a role in mediating microRNA transfer (41). First, according to Moon et al., the intravenous administration of MSC-EVs resulted in the stimulation of blood vessel formation (angiogenesis) and the generation of new nerve cells (neurogenesis) within 24 h after inducing MCAO. This effect was found to be positively associated with the dosage of MSC-EVs (41). The levels of miR-184 and miR-210 in MSC-EVs were higher than those in fibro EVs (41). Transfecting neural stem cells and human umbilical vein endothelial cells with miR-184 and miR-210 may enhance their proliferation. This means that miR-184 and miR-210 may be used by MSC-EVs to help vascular endothelial cells and neural stem cells multiply after ischemic stroke (41). Secondly, MSCs-derived exosome miR-455-3p targeted PDCD7 to alleviate hippocampal neuronal injury in MCAO/R-treated mice and injury of OGD/R-treated Neuro-2a cells (42). Furthermore, the experimental results of Geng et al. showed that MSC-EVs overexpressing miR-126 significantly increased the number of doublecortin positive and von Willebrand factor positive cells compared with normal exosomes (43), which suggests that miR-126 may be involved in EV-mediated angiogenesis and neurogenesis. Finally, MSC-EVs may play an indirect role in nerve repair after ischemic stroke. *In vitro* experiments by Xin et al. showed that MSC-EVs overexpressing miR-133b could increase the secretion of exosomes by astrocytes, while the latter could significantly increase the number and length of axons (44).

### 3.2 MSCs and Alzheimer’s disease

AD is characterized by increased deposition of *β*-amyloid peptides and aggregation of hyperphosphorylated tau in NFT (45). However, clinical symptoms vary with the region of brain injury. Typical clinical symptoms include progressive decline of episodic memory and executive functions (46). Recently, MSCs have garnered significant interest as possible cell-based therapeutic tools because of their capacity to migrate and facilitate damage repair. MSCs promote the restoration of neurological function and the formation of new blood vessels by releasing neurotrophins and proteins that regulate angiogenesis (47, 48). Next, we summarize the effects of MSCs during AD treatment.

#### 3.2.1 Inhibiting neuroinflammation

The pathological mechanism of AD is known to be active inflammatory reactions, and studies have shown that MSCs transplantation can alleviate brain inflammation by regulating the secretion of inflammatory and therapeutic factors (49). In a senescence-accelerated mouse-prone animal model, Li et al. injected BM-MSCs into bone marrow cavities and demonstrated beneficial effects. The levels of inflammatory cytokines (IL-1*β*, IL-6, iNOS, and HO-1) were diminished, while the concentration of TGF-β (a therapeutic cytokine) was augmented, resulting in an amelioration of the inflammatory condition, a reduction in oxidative stress, and an enhancement of cognitive function (50). When human umbilical cord blood-derived mesenchymal stem cells (hUCB-MSCs) were transplanted into the brains of mice with the APP/PS1 genetic mutation, the activity of microglia was reduced, resulting in a decrease in the production of inflammatory substances and an increase in the release of therapeutic substances, thereby improving the inflammatory condition (49). In a study involving the transplantation of human menstrual blood-derived stem cells (MenSCs) into the cerebellum of an APP/PS1 mouse model, it was seen that these cells stimulated the activation of microglia. This activation led to the secretion of anti-inflammatory substances through an alternative neuroprotective phenotype (51). Redondo-Castro et al. demonstrated when microglial BV2 cells were activated with bacterial lipopolysaccharide (LPS) and then treated them with BM-MSCs, BM-MSCs-treatment upregulated the expressions of anti-inflammatory and neuroprotective factors (IL- 10, VEGF, BDNF, G-CSF, NGF and IL-1Ra) (52). A rat model of AD was given MSCs through the nose. These cells lowered levels of inflammatory factors (IL-1β, IL-12, TNF-*α* and IFN-*γ*) in the hippocampal area (53).

#### 3.2.2 Microglia changes

Reactive microglia play a role in removing proteins from the brain during chronic inflammation. However, their limited surface area hinders their ability to take up extracellular proteins and remove them. Furthermore, reactive microglia stimulate astrocytes to assume detrimental reactive states and enhance oxidative stress through the activation of neurotoxic oxygen and nitrogen compounds (54). When Aβ accumulates in the brain, microglia try to restore brain functions by absorbing proteins and secreting anti-inflammatory factors. However, chronic microglial activation increases microglia numbers and the expression of inflammatory factors and decreases protein clearance. Several authors have documented that administering stem cell injections in animal models of AD suppresses the activation of microglia and decreases the levels of inflammatory factors in the brain (55–58). When BM-MSCs were transplanted into an APP/PS1 mouse model of AD via tail vein, microglial numbers in the cortex, microglia sizes, and pro-inflammatory factor levels (TNF-*α* and IL-6) were reduced (55). Also, putting Wharton’s jelly-derived MSCs (WJ-MSCs) into an APP/PS1 AD mouse model through the tail vein decreased the number of reactive microglia and levels of pro-inflammatory factors (IL-1*β* and TNF-α) while increasing levels of IL-10, an anti-inflammatory factor (56). Furthermore, expressions of inflammatory factors were downregulated, an alternative activated phenotype of microglia was induced, and phagocytosis was improved in primary rat microglia cultured in rat MSCs conditioned media. In addition, TGF-*β* secreted by MSCs blocked the nuclear factor-κB pathway and restored the TGF-β pathway (57). Cho et al. reported when human placenta-MSCs (pMSCs) were intracerebroventricularly or intravenously injected into an AD rat model, AD-associated increases in microglia numbers in brain lesion rapidly returned to normal levels (58).

#### 3.2.3 Amyloid-β removal

The Aβ plaque is a representative characteristic of AD. Stem cell transplantation has been shown to reduce Aβ plaque levels by reducing microglia numbers, activating proteasomes, and enhancing autophagy and β-amyloidase secretion (59–66). β-amyloidase exhibits Aβ -degrading activity such as insulin degrading enzyme. In detail, agouti-related peptide secreted by MSCs increased proteasome activity and decreased Aβ plaque (59). Soluble intracellular adhesion molecule-1 (sICAM-1) secreted by hUCB-MSCs induced the expression of neprilysin (a Aβ -degrading enzyme) (60). Growth differentiation factor-15 (GDF-15) secreted by hUCB-MSCs was found to reduce Aβ plaques and promote hippocampal neurogenesis (61). Furthermore, thrombospondin-1 (TSP-1) secreted by hUCB-MSCs restored neuronal synaptic density impairment caused by Aβ (62). Yang et al. transplanted hUCB-MSCs-derived neuron-like cells into bilateral hippocampus in Aβ-induced AD model and observed they activated microglia, promoted the secretions of anti-inflammatory substances, reduced Aβ accumulation, and restored memory deficits (63). In a mouse model of acute AD, bilateral hippocampus injections of BM-MSCs removed Aβ by activating microglia (64). Shin et al. observed intravenous administration of MSCs improved lysosome-autophagy and removed Aβ by activating autophagy-associated LC3-II and BECN1/Beclin 1 (65). When traumatic brain injury was induced, Aβ plaques aggregated in the mouse model. pMSCs were intravenously double injected at 4 and 24 h post-injury onto the traumatic brain injury mice model, and reduced infarct sizes, inflammatory, oxidative responses, and inhibition of Aβ plaque formation were observed (66). Aβ-induces tau hyperphosphorylation, which leads to cytotoxicity and cell death, forming NFT. Studies have shown that numerous stem cell therapies can prevent tau hyperphosphorylation-induced cytotoxicity (49, 51, 67). Zilka et al. used rat MSCs, or substances released by them to treat cells that had mutated tau proteins. This decreased cell death and increased metabolic activity, but did not change the expression of tau. The authors proposed that MSCs can lessen the cytotoxic effect of modified tau in AD (67). We observed rapid reductions of tau hyperphosphorylation, increases in anti-inflammatory factor levels, and changes in microglial phenotype when we transplanted hUCB-MSCs or MenSCs into the cerebellum of the APP/PS1 mouse model (49, 51). Trypsin-like activity (proteolytic activity) of proteasomes is significantly lower and the activity of immunoproteasomes is significantly higher in neurons from AD patients than in those of normal subjects (59). To recover ubiquitin proteasome system function in AD, Lee et al. administered agouti-related peptide secreted from MSCs or WJ-MSCs to mouse hippocampi and observed a significant increase in proteasome activity and a decrease in ubiquitin-conjugated protein accumulation (59).

#### 3.2.4 Functional recovery of autophagy

Autophagy is typically responsible for maintaining cellular homeostasis by breaking down unneeded or aberrant cellular components. However, when autophagy malfunctions, it can contribute to the development of cancer, inflammation, and neurological disorders. Several authors have documented that the administration of stem cell injections in mouse models of AD suppresses the activation of microglia and decreases the levels of inflammatory factors in the brain (68). Interestingly, Guan et al. showed autophagy plays an important role in maintaining stemness, stem cell expansion, and differentiation (69). Another study performed by Salemi et al. noted autophagy levels were elevated in human skin and blood-derived MSCs and important for maintaining stemness (70). Furthermore, the activation of autophagy via Bcl-xL is known to promote MSCs survival and differentiation (71).

There have been relatively few investigations conducted on the correlation between autophagy and AD. Wang et al. conducted a study where they injected MSCs into the tail vein of rats with vascular dementia. They observed a large rise in the levels of autophagy proteins LC3-II and Beclin-1. This suggests a strong association between autophagy and AD (72). After the transplantation of MSCs, there was a decrease in synaptic damage, mitochondrial aggregation, and damage to the presynaptic area. Additionally, there was an increase in the expression of BDNF and N-methyl-D-aspartate receptor 1 in the hippocampus, resulting in enhanced cognitive function (72). When MSCs were injected through the tail vein in a mouse model of AD, it significantly enhanced autolysosome formation and Aβ clearance (65).

#### 3.2.5 BBB function recovery

Under normal conditions of the BBB, medicines can be transported to the brain through carrier-mediated transport or receptor-mediated transcytosis. Nevertheless, diverse dysfunctions of the BBB are identified in the cortex and hippocampus of patients with AD. Examples are capillary leakage and infiltration of blood cells, pericyte degeneration, endothelial degeneration, and micro vessel reduction and shortening (73). The breakdown of the BBB results in the buildup of harmful substances that are toxic to the nervous system. This breakdown also triggers the activation of astrocytes and microglia, as well as an inflammatory response. Consequently, there is an accumulation of cellular debris, including pericyte and endothelial cells, which hinders the absorption of drugs and the delivery of therapeutic treatments to the brain (73).

MSCs and induced pluripotent stem cells (iPSCs) have been differentiated into endothelial cells to recover BBB functions, and this differentiation has shown to play key roles in the regeneration of blood vessels. MSCs can be differentiated into endothelial cells with differentiation media containing vascular endothelial growth factor (VEGF), basic fibroblast growth factor (bFGF), insulin like growth factor (IGF), epidermal growth factor (EGF), ascorbic acid, and heparin (74). In an APP/PS1 mouse model, BM-MSCs transplantation increased VEGF expression levels and improved endothelial dysfunction and synaptic plasticity (75). Moreover, several studies have successfully used stem cells to induce vascular regeneration and functions of vascular endothelial cells for treating ischemic diseases, and this technique could be useful for treating AD.

## 4 Approaches to enhance therapeutic effects of MSCs

Current routes of administration include intravenous, intraventricular, and nasogastric, but the timing of administration remains to be explored. Although MSCs represent a promising candidate for CNS regeneration, low therapeutic efficacy limits their clinical use. Different cultural conditions may alter MSCs’ survival, homing, and key functional features. It was found by Madrigal et al. that growing cells in low-oxygen environments might have therapeutic effects on MSCs by increasing the production of HGF, TGF-b, VEGF, and TSG-6, all of which are important for CNS regeneration (76). Others demonstrated that pro-inflammatory stimuli and tri-dimensional growth stimulate trophic factors secretion of MSCs (77). It is evident that cultural conditions will considerably affect the therapeutic efficacy of MSCs. Apart from culture medium, developed therapeutic strategies may also enhance the therapeutic effects of MSCs, such as delivery route and timing. Although there is no consensus on the optimum delivery route of MSCs, intracerebroventricular transplantation may be the most efficacious. Park et al. found that intracerebroventricular MSC transplantation may be associated with endogenous enhancement, compared to intravenous and intraparenchymal routes for CNS regeneration, after reviewing previous pre-clinical and clinical studies (78). The intracerebroventricular transplanted MSCs attenuated brain injury in a time-dependent manner. Significant neuroprotection was demonstrated when administered from 2 to 7 days after induction in intraventricular hemorrhage rat models (79).

## 5 Safety of MSC transplantation

Although using MSCs in animal stroke models was generally safe and had a significant effect on behavioral outcomes, some studies still showed side effects such as embolism infection, and tumor formation (80, 81). Aβ accumulation and calcium in the thalamus also appear (82). Research on rat stroke models suggested that intra-arterial (IA) MSCs delivery can reduce the flow of middle cerebral artery (MCA). However, this side effect appears to be dose dependent. A dosage of 1 × 10^5^ MSCs was shown to be the maximal tolerable dose of IA infusion, making no concessions to the blood flow of MCA. One study also showed that delivering MSCs at 24 h after stroke significantly improved neurological function and reduced the infarct size at 1 month compared with control but delivering 1 h after stroke did not confer such protective effects (83). Wang et al. found that there was no standard dose for stem cell therapy currently associated with the route of administration and disease types. For intracerebral parenchymal transplantation, an excessively large transplant dose affected the nutrition of transplanted cells and could cause micro emboli and vascular occlusion when administered intravascularly (84). Although there is no uniform dose standard, dose control is very important in preventing embolism. Intravenous infusion is thought to be associated with embolization, and embolization can be reduced by intraperitoneal or other routes of transplantation (85).

Many safety problems have emerged with the intracerebral transplantation and interventional neuroradiography in acute stroke settings, such as maintain biological stability of the therapeutic product, larger MSCs doses can potentially affect organ perfusion, and the safety of allogeneic MSCs (80). Another investigation demonstrated that amyloid-beta and calcium accumulation in the thalamus occurred following the intravenous administration of human bone marrow mesenchymal stem cells (MSCs) in a rat model of MCAO. Quantitative analysis revealed a markedly significant increase in amyloid-beta and calcium deposits in the thalamus 48 h post-MSC infusion. Furthermore, a distinct correlation was observed between diminished forelimb performance on postoperative day 42 and the accumulation of amyloid-beta and calcium in the thalamus (82). MSCs transplantation animal experiments found no obvious immune rejection. However, studies had shown that *in vitro* licensed WJMSCs did not improve experimental autoimmune encephalomyelitis in rats, due to increased immunogenicity resulting in rapid rejection (86).

## 6 Clinical trials of MSCs transplantation

Cells derived from bone marrow displayed great prospects for safety and initial efficacy (87, 88). Some clinical tests in Phase I and Phase II have already begun, using cell populations originating from MSCs (Table 3). Early results revealed that intravenous injection of MSCs did not give raise to significant adverse effects but could improve functional measurements such as the Barthel Index (BI), the National Institutes of Health Stroke Score (NIHSS) and the modified Rankin Scale (MRS) (87, 89). A long-term follow-up study of intravenous autologous MSCs transplantation in patients with ischemic stroke showed that no significant side effects were observed, and the follow-up MRS score was decreased compared with the control group (8). A meta-analysis from Lalu et al. suggested that MSC therapy appeared safe, but there was a significant association between MSCs, and transient fever based on the current clinical trials, so further larger scale controlled clinical trials with rigorous reporting of adverse events were required to further define the safety profile of MSCs (90). Contradictory data shows that MSC injection may not improve the results of the function (91). These studies used autologous MSCs which were expanded in culture before MSC transplantation (8, 87–91). Although no side effects of the products were reported, the cells were amplified in autologous serum, leading to faster cell expansion and reducing concern of heterogeneous contamination.

**Table 3:** MSCs transplantation in nervous system disease trials.

NCT	Conditions	Country	Phase	Cell source	Status
01091701	Stroke	Malaysia	I/II	MSCs/Allogenicr	Withdrawn
04093336	Stroke	China	I/II	MSCs/Autologous	Recruiting
04907188	Stroke	American	I	ADSCs/Autologous	No longer available
05008588	Stroke	Indonesia	I	UC-MSCs/Autologous	Recruiting
01461720	Stroke	Malaysia	II	BMSCs/Autologous	Unknown
06518902	Stroke	American	I	UC-MSCs/Autologous	Not yet recruiting
03356821	Stroke	Netherlands	ND	BMSCs/ Allogenicr	Completed
02611167	PD	American	ND	BMSCs/ Allogenicr	Completed
03684122	PD	Jordan	ND	UC-MSCs/Autologous	Unknown
03550183	PD	China	ND	UC-MSCs/ Allogenicr	Unknown
01446614	PD	China	I/II	BMSCs/Autologous	Unknown
04506073	PD	American	II	BMSCs/ Allogenicr	Completed
04772378	PD	American	All	ADSCs/Autologous	No longer available
04388982	AD	China	I/II	ADSCs/ Allogenicr	Unknown
03172117	AD	Korea	I/II	UC-MSCs/Autologous	Completed
02054208	AD	Korea	I/II	UC-MSCs/Autologous	Completed
01547689	AD	China	All	UC-MSCs/Autologous	Unknown
04954534	AD	Korea	ND	UC-MSCs/Autologous	Unknown
01696591	AD	Korea	ND	UC-MSCs/Autologous	Unknown

## 7 Conclusion and future perspective

This review proposes a novel method to explore the therapeutic effects of MSCs on neurogenesis through scientometric analysis geared towards identifying new research hotspots. An in-depth and comprehensive review has been performed based on the results of the scientometric study. This is the first review to summarize the therapeutic impacts of MSCs on stroke and AD, thus providing a novel unique perspective for their treatment. It is important to elucidate the mechanisms underlying the regulatory role of MSCs in NSC proliferation and differentiation associated with psychiatric and neurological disorders, including stroke and AD accompanied by deterioration of neurogenic signaling pathways and factors implicated in adult neurogenesis. Unraveling the general profile of complex signaling pathways in different neurological disorders improves the understanding of the functional significance of MSCs.

However, our article also has some limitations: 1. Incomplete database coverage: Bibliometrics relies on Web of Science, but these databases have regional, linguistic, and disciplinary biases; 2. The issue of using quantity instead of quality: Common indicators in bibliometrics (such as citation count and H-index) reflect more “influence” than “quality.” High citation may be due to controversy or negative citations rather than academic value. 3. Ignoring innovation: Lagging citations may underestimate early or disruptive research, while high short-term citations may overestimate trend-following research.

Although mesenchymal stem cells have shown enormous potential in fields such as neurogenesis, their clinical application still faces many challenges, especially the selection of the optimal dose and administration time. The following is the analysis of these two major challenges and possible solutions: The dose-effect relationship is not clear: 1. The dose does not linearly relate to the therapeutic effect of MSCs. Excessive doses may cause side effects (such as immune rejection and pulmonary embolism), while insufficient doses may be ineffective. The dosage requirements vary significantly among different disease types (such as myocardial infarction, osteoarthritis, and graft-versus-host disease); 2. Heterogeneity effect: Differences in the source of MSCs (bone marrow, fat, umbilical cord, etc.), culture conditions, and batches may lead to unstable therapeutic effects and make it difficult to standardize the dosage uniformly. Solution strategy: 1. Dose exploration based on preclinical studies. 1.1 Establish a dose gradient experiment in the animal model and determine the effective window in combination with pathophysiological characteristics (such as injury range and inflammation level); 1.2 Predict the survival, distribution, and mechanism of action of cells *in vivo* through pharmacokinetic/pharmacodynamic models. 2. Individualized dose adjustment. The dosage is dynamically adjusted based on parameters such as the patient’s weight, the severity of the disease, and the immune status. For example, critically ill patients may require higher doses or multiple infusions. 3. New delivery technologies enhance efficiency. 3.1 Use biomaterial scaffolds or 3D microcarriers to prolong the local retention time of cells and reduce dose requirements; 3.2 Enhance the survival rate and function of MSCs through gene editing (such as overexpression of anti-apoptotic genes) or pretreatment (such as hypoxic culture) and reduce the effective dose. The issue of administration time. 1. Narrow treatment time window: The response to MSCs varies greatly at different disease stages (such as the acute stage vs. the chronic stage). For example, a premature infusion after a myocardial infarction may cause cell death due to the inflammatory microenvironment, while a too- late infusion may miss the repair opportunity. 2. The dynamic microenvironment affects the therapeutic effect: Inflammatory factors, hypoxia, oxidative stress, and others in the local microenvironment may inhibit the survival and function of MSCs. It is necessary to choose a time point when the microenvironment is relatively stable for administration. Solution strategy: 1. Implement a sequential intervention strategy that is based on the mechanisms of the disease. Biomarkers, such as inflammatory factor levels and imaging features, dynamically monitored the disease process to determine the optimal intervention time window. For example: Neurodegenerative diseases: Intervention in the early stage when neurons have not undergone extensive apoptosis; 2. Staged combined treatment. First, the microenvironment is regulated through drugs or biological agents (such as inhibiting excessive inflammation), and then MSCs are infused to improve their survival rate and function. 3. Real-time monitoring and feedback. Develop non-invasive imaging techniques (such as magnetic particle imaging and fluorescence labeling) to track the *in vivo* distribution of MSCs and dynamically adjust the administration time in combination with therapeutic markers.

We believe that future research directions mainly fall into two aspects: 1. Mechanism-driven research. Identify the target sites of MSCs in specific diseases (such as paracrine factors and exosomes) and develop alternative therapies (such as extracellular vesicles) to reduce the reliance on intact cells. 2. Dynamic control technology. Design “intelligent responsive” MSCs and activate their functions at specific times or in a microenvironment through optogenetic or drug-induced systems.

## Data Availability

The raw data supporting the conclusions of this article will be made available by the authors, without undue reservation.
